# Volume and T2 relaxation time measurements of quadriceps femoris and hamstring muscles are reliable and reproducible

**DOI:** 10.3906/sag-2101-49

**Published:** 2021-08-30

**Authors:** Şerife Şeyma TORĞUTALP, Ömer ÖZKAN, Şafak PARLAK, Kader Karlı OĞUZ, Feza KORKUSUZ

**Affiliations:** 1 Clinic of Sports Medicine, Gaziler Physical Therapy and Rehabilitation Training and Research Hospital, Ankara Turkey; 2 Department of Sports Medicine, Faculty of Medicine, Hacettepe University, Ankara Turkey; 3 Department of Radiology, Faculty of Medicine, Hacettepe University, Ankara Turkey

**Keywords:** Magnetic resonance imaging, reliability, reproducibility, skeletal muscle, thigh

## Abstract

**Background/aim:**

Volume and T2 relaxation time measurements of the skeletal muscle provide quantitative information. We aimed to evaluate the interobserver reliability and the intraobserver reproducibility of measurements of volumes and T2 relaxation times of the quadriceps femoris and the hamstring muscles.

**Materials and methods:**

A cross-sectional reliability study was conducted on ten recreational athletes. The images of the quadriceps and the hamstring muscles of both limbs were obtained using a 3.0 Tesla magnetic resonance imaging (MRI) scanner. Two sports medicine specialists measured muscle volumes from a total of 2560 images and T2 relaxation times from a total of 40 images, and repeated this once more. The intraobserver and interobserver compliance were assessed by the intraclass correlation coefficient (ICC) and Cronbach’s alpha (α).

**Results:**

Volume and T2 relaxation time of quadriceps femoris and hamstring muscle measurements with MRI had good to excellent reliability (Muscle volume; intraobserver ICCs: between 0.97 and 0.99, α: between 0.98 and 0.99 and interobserver ICCs: between 0.96 and 0.99, α: 0.99. T2 relaxation time; intraobserver ICCs: between 0.74 and 0.96, α: between 0.85 and 0.98 and interobserver ICCs: between 0.75 and 0.90, α: between 0.85 and 0.95).

**Conclusion:**

Volume and T2 relaxation time measurements of the quadriceps femoris and the hamstring muscles are reliable and reproducible.

## 1. Introduction

Skeletal muscle is the largest lean tissue in humans and constitutes about half of the total body mass [1]. Skeletal muscle is vital for metabolic health in addition to its importance in movement [1]. Morphometric data of skeletal muscle may allow evaluating the changes regarding age, sex, injury, and illness [2]. Changes in skeletal muscle volumes and shapes may occur in physiological conditions such as due to strengthening program and aging, as well as pathological conditions such as injury and neuromuscular diseases [3–6]. Muscle volume measurement is accordingly used to evaluate the diagnosis and monitoring of neuromuscular diseases and the effectiveness of treatment [7]. Additionally, it helps the assessment of anatomical and structural features, which are the cornerstone of the athletic performance, especially in sports medicine [8].

MRI is accepted as a gold standard and validated method to evaluate skeletal muscle mass for clinical and research purposes, and provides an accurate assessment of the change of skeletal muscle mass over time [7,9,10]. The advantages of MRI are the absence of radiation exposure and the ability to provide multiplanar and volumetric 3D imaging, hence the measurement of regional muscle cross-sectional area and muscle volume estimation [11]. Additionally, MRI provides quantitative information on skeletal muscle quality [8,12]. T2 relaxation time mapping (T2 mapping), which is a quantitative MRI technique, has been used as an advanced method to detect the microstructure and the perfusion of the skeletal muscle [8,12]. Most studies analyzing the cross-sectional area and T2 relaxation times of the quadriceps femoris and hamstring muscle mainly investigated validity, however, reliability has rarely been reported as part of research with a limited number of participants [8, 13, 14].

Rapid skeletal muscle loss might be seen especially in cases such as limb immobilization after injury [15]. On the contrary, the increase in skeletal muscle mass causes an increase in strength, power, and athletic performance [16]. Therefore, the evaluation of skeletal muscle mass is important in sports medicine practice with respect to follow-up of athletes and return to play after injury. In this study, we asked whether the quantitative measurements of thigh muscles by MRI are reliable and reproducible. Specifically, we aimed to evaluate the interobserver reliability and intraobserver reproducibility of the volume and T2 relaxation time measurements of the quadriceps femoris and the hamstring muscles with 3.0 Tesla MRI.

## 2. Materials and methods

### 2.1. Design

A cross-sectional study was planned on recreational athletes, who applied to the sports medicine outpatient clinic for preparticipation physical evaluation in the 2019–2020 season. 

### 2.2. Participants

A total of 10 volunteers (5 females, 5 males) who were considered healthy based on their history and preparticipation physical evaluation were included. Baseline characteristics of the participants are given in Table 1. Mean age of the participants was 25.9 ± 3.6 (range 21–32) years old. All subjects were recreational runners at least 30 min per day and 5 times per week. Participants were excluded if they reported: conditions such as using an implantable cardioverter-defibrillator and implantable nerve stimulator as they create contraindication for MRI; using medication such as statin or supplement that affects muscle health; having diabetes, renal or liver failure, neuromuscular or inflammatory disease; having rheumatic musculoskeletal disorders such as rheumatoid arthritis, spondyloarthritis, osteoarthritis or gout; having a history of musculoskeletal injury and surgery involving the areas to be measured. Individuals who could not be imaged for any reason other than abovementioned were also excluded.

**Table 1:** Baseline characteristics of the participants.

N = 10	Mean ± SD	Median (min-max)
Age, years	25.9 ± 3.6	26 (21–32)
Height, cm	171.9 ± 9.7	167.5 (160–185)
Weight, kg	66.7 ± 3.5	67 (50–81)
BMI, kg/m2	22.4 ± 2.1	22.7 (19.1–26.1)
Fat %	16.3 ± 6.7	13.6 (8.2–28.3)
Leg muscle mass, kg		
Right	9.1 ± 1.9	9.1 (6.7–11.6)
Left	8.9 ± 1.9	8.8 (6.6–11.1)
Total	18.0 ± 3.9	17.9 (13.3–22.7)

BMI: body mass index, cm: centimeter, kg: kilogram, m: meter, max: maximum, min: minimum, SD: standard deviation.

The question “If you would shoot a ball on a target, which leg would you use to shoot the ball?” was asked to determine the leg dominance of the participants, and identified themselves as right-side-dominant [17]. 

### 2.3. Anthropometric measurements

The participant’s height and weight were measured using a digital scale (Seca 769, Hamburg, Germany), and BMI was calculated by dividing their body weight by height squared (kg/m^2^). 

Body composition including total fat percentage and regional lean mass was assessed with BIA (BC 418, Tanita Corporation of America, Inc., Illinois, USA). 

### 2.4. Magnetic resonance imaging, segmentation, volume estimation, T2 relaxation time

Healthy participants, who did not meet the abovementioned exclusion criteria, underwent an MRI protocol for muscle imaging and T2 mapping. Images were obtained after participants laid supine for a minimum of 10 min as fluid shift might affect volume measurements [18]. Imaging of the quadriceps and hamstring muscles of both limbs was obtained using a 3.0 Tesla MRI scanner (MAGNETOM Trio, A Tim System, Siemens Medical Solutions, Erlangen, Germany) settled in National Magnetic Resonance Research Center (UMRAM, Ankara, Turkey). The imaging protocol was as follows: T1-weighted imaging in the coronal plane involving the respective muscles (TR/TE: 1070/10ms, FOV: 450 mm, matrix: 384 × 384, slice thickness: 5 mm); axial Dixon 3D T1-vibe (TR/TE: 5.27/2.45 ms, FOV: 380 mm, matrix: 320 × 240, slice thickness: 4 mm) which fat and muscle tissue can be distinguished. These images could be obtained in two parts consecutively due to length of these muscles and provided visualization of both the hip and the knee joints. Axial T2 mapping imaging (TR: 1000, TE: 13.8 ms, FOV: 380 mm, matrix: 320 × 320, slice thickness: 10 mm, NEX: 2.0, FA: 180°) was centered on midfemur level and was obtained for 48 mm length (5 sections) from superior to inferior for each participant.

**Figure 1:**
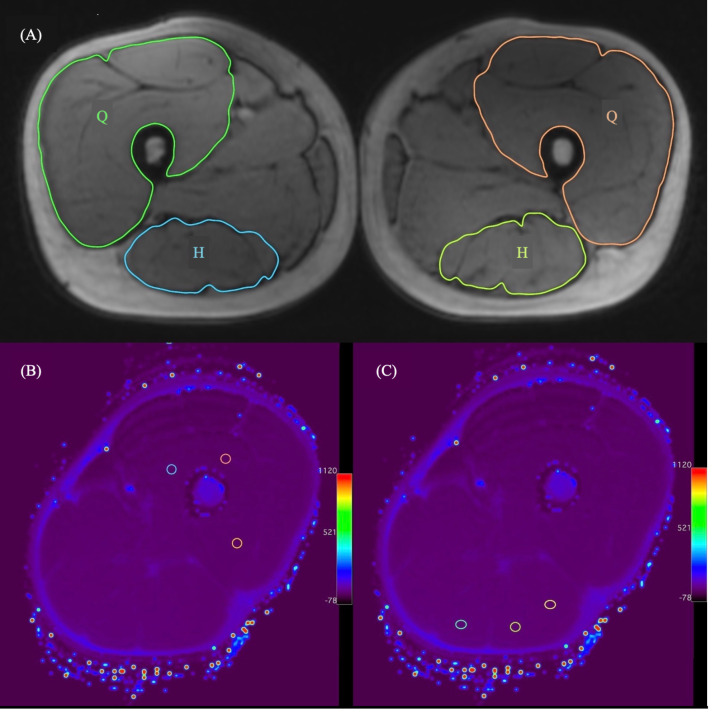
(a) Determination of the right and left quadriceps femoris (Q) and hamstring (H) muscles’ boundaries on the 3D T1-vibe axial image. (b) Measurement of T2 relaxation time of the left quadriceps muscle. (c) Measurement of T2 relaxation time of the left hamstring muscle. T2 relaxation time was calculated from the mean of three region of interests (ROI) with a diameter of 0.2 cm2 for the quadriceps and hamstring muscles. Each ROI was selected to avoid any visible blood vessels or fat. cm: centimeter, H: hamstring muscle, Q: quadriceps muscle.

The volumes of the muscles were computed by a software (OsiriX Lite v.11.0.2, Osirix Foundation, Geneva, Switzerland) by manually drawing outlines of anatomical cross-sectional area for the quadriceps and the hamstring muscles on axial 3D Dixon T1-vibe images for each section from the lateral knee joint line to the hip joint center (Figure 1a) [19]. 

T2 relaxation times of the quadriceps and the hamstring muscles were also measured on T2 mapping imaging using the same software (OsiriX Lite v.11.0.2, Osirix Foundation) at the midfemur level. T2 relaxation times were measured from three different regions in each muscle at the selected level using same size region of interest (ROI) (0.2 cm^2^) and the mean of these values was accepted T2 relaxation time of each muscle (Figures 1b and 1c). 

### 2.5. Measurements of muscle volumes and T2 relaxation times by sports medicine specialists

All of the abovementioned measurements were performed by two sports medicine specialists. Both had received training at different time points. The training was given by a radiologist with more than 6 years of experience in MRI muscle volume assessment. Each of the sports medicine specialists measured muscle volumes from a total of 2560 images and T2 relaxation times from a total of 40 images at baseline and repeated this 15 days later. All images were anonymized and the sports medicine specialists were blinded to the participants. 

### 2.6. Statistical analysis

Statistical analyses were performed using the R version 3.6.2. The variables were investigated using visual (histograms and probability plots) and analytical methods (Shapiro–Wilk test) to determine normal or non-normal distributions. Descriptive analyses were presented using means, standard deviations (SDs), medians, minimums, and maximums. Intraobserver reproducibility and interobserver reliability were calculated with a 95% confidence interval (CI). The intraobserver and interobserver compliance were assessed by the intraclass correlation coefficient (ICC) score, Cronbach’s alpha (α) and Bland–Altman plots. The ICC scores were interpreted as follows: < 0.40: poor clinical significance; 0.40–0.59: fair clinical significance; 0.60–0.74: good clinical significance, > 0.75: excellent clinical significance [20].

## 3. Results

3.1. Volume and T2 relaxation time measurements of quadriceps femoris and hamstring muscles

The mean quadriceps femoris and hamstring muscle volumes and T2 relaxation times of muscles, which were measured by two sports medicine specialists at two time points, are presented in Table 2.

**Table 2:** Volume and T2 relaxation time values of the quadriceps femoris and the hamstring muscles measured by two sports medicine specialists at two time points.

Specialist	Timepoint	Quadriceps femoris muscle				Hamstring muscle			
		Muscle volume(cm3)		T2 relaxation time (ms)		Muscle volume(cm3)		T2 relaxation time(ms)	
		Right	Left	Right	Left	Right	Left	Right	Left
1	1	1826.0 ± 336.5	1802.9 ± 342.6	33.7 ± 2.2	32.1 ± 3.5	744.4 ± 173.6	714.6 ± 140.3	37.3 ± 1.8	39.8 ± 2.4
	2	1800.1 ± 313.6	1796.9 ± 349.1	33.4 ± 1.7	31.6 ± 3.1	729.1 ± 171.2	705.7 ± 140.8	37.6 ± 2.3	39.6 ± 2.5
2	1	1809.9 ± 316.1	1789.5 ± 346.9	33.1 ± 1.7	31.9 ± 2.5	760.1 ± 160.1	732.9 ± 139.6	37.5 ± 1.9	39.2 ± 1.7
	2	1809.7 ± 315.2	1825.3 ± 355.1	33.6 ± 2.6	31.5 ± 2.6	766.6 ± 178.5	738.7 ± 144.5	37.3 ± 1.9	39.7 ± 2.2

### 3.2. Intraobserver and interobserver compliance

Quadriceps femoris and hamstring muscle volume and T2 relaxation time measurements with MRI had good to excellent reliability (Muscle volume; intraobserver ICCs: between 0.97 and 0.99, α: between 0.98 and 0.99 and interobserver ICCs: between 0.96 and 0.99, α: 0.99. T2 relaxation time; intraobserver ICCs: between 0.74 and 0.96, α: between 0.85 and 0.98 and interobserver ICCs: between 0.75 and 0.90, α: between 0.85 and 0.95) (Table 3). Intraobserver reliabilities were excellent for both sports medicine specialists regarding all measurements (ICCs ranging from 0.79 to 0.99), except for T2 relaxation time of the right quadriceps muscle, which was measured by Specialist-2 (ICC: 0.74). Interobserver reliabilities were also excellent for both muscles in terms of muscle volume and T2 relaxation time (ICCs ranging from 0.75 to 0.99).

**Table 3:** Reliability results of volume and T2 relaxation time measurements of the quadriceps femoris and the hamstring muscles performed by two sports medicine specialists at two time points.

			Intraobserver						Interobserver		
			Specialist 1			Specialist 2					
			ICC	CI	α	ICC	CI	α	ICC	CI	α
Quadricepsfemorismuscle	R	Muscle volume (cm3)	0.99	0.95–0.99	0.99	0.99	0.99–1.00	0.99	0.99	0.98–0.99	0.99
		T2 relaxation time (ms)	0.79	0.37–0.94	0.88	0.74	0.27–0.93	0.85	0.83	0.61–0.93	0.90
	L	Muscle volume (cm3)	0.99	0.96–0.99	0.99	0.99	0.94–0.99	0.99	0.99	0.98–0.99	0.99
		T2 relaxation time (ms)	0.86	0.54–0.96	0.92	0.84	0.51–0.96	0.91	0.75	0.47–0.89	0.85
Hamstringmuscle	R	Muscle volume (cm3)	0.98	0.91–0.99	0.99	0.99	0.96–0.99	0.99	0.97	0.81–0.99	0.99
		T2 relaxation time (ms)	0.93	0.75–0.98	0.96	0.92	0.73–0.98	0.96	0.90	0.77–0.96	0.95
	L	Muscle volume (cm3)	0.97	0.89–0.99	0.98	0.99	0.95–0.99	0.99	0.96	0.79–0.99	0.99
		T2 relaxation time (ms)	0.96	0.85–0.99	0.98	0.91	0.52–0.98	0.97	0.90	0.77–0.96	0.95

α: Cronbach’s alpha, cm: centimeter, CI: confidence interval, ICC: intraclass correlation coefficient, L: left, ms: millisecond, R: right.

**Figure 2:**
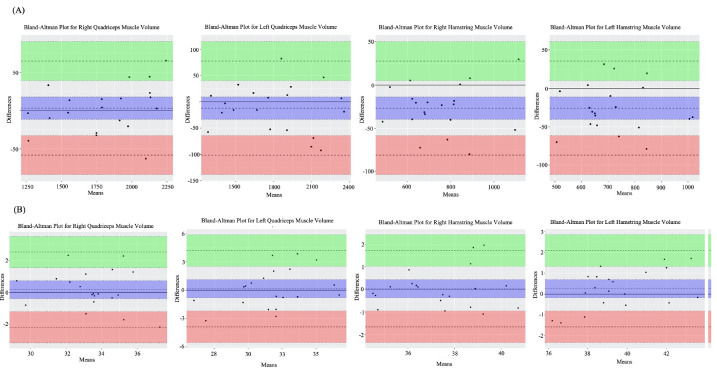
Bland–Altman plots of interobserver variability. (a) Quadriceps femoris and hamstring muscles’ volumes. (b) Quadriceps and hamstring muscles’ T2 relaxation times. The middotted line shows the average of the differences. The upper and lower dotted lines show 2 standard deviations above and below the mean

Both intraobserver and interobserver ICC scores of the muscle volume measurements were higher in comparison to the T2 relaxation time measurements. Bland–Altman plots of interobserver variability in the measurement of quadriceps femoris and hamstring muscle volumes and T2 relaxation times are presented in Figures 2a and 2b, whereas Bland–Altman plots of intraobserver variability are shown in Figures 3a–3d.

## 4. Discussion

Skeletal muscle mass is important for human health, performance and functional capacity. Recent studies have shown that MRI provides an accurate assessment of the change of muscle mass over time [7,9]. Accordingly, in this study, we aimed to evaluate the reliability and the reproducibility of the volume and T2 relaxation time measurements of the quadriceps femoris and the hamstring muscles through images obtained using a 3.0 Tesla MRI. Our results showed good to excellent intra- and inter-observer reliability in measures of the quadriceps femoris and the hamstring muscle volumes and T2 relaxation times (ICC ranging from 0.74 to 0.99). Based on the conclusion that the volume and T2 relaxation time measurements from images obtained with MRI were reliable and reproducible to determine the quantity and quality of the quadriceps femoris and the hamstring muscles, we recommend that they can be used in clinical practice and future studies.

We found that intra- and interobserver reliability was excellent for cross-sectional area measurements of the quadriceps femoris and the hamstring muscles determined from 4 mm interslice distance (ICCs ranging from 0.96 to 0.99). Barnoin et al. aimed to propose a manual segmentation method for individual quadriceps femoris muscles and to test its interrater reliability for muscle volume estimation [21]. They measured muscle anatomical cross-sectional areas on 5-mm thickness axial images reconstructed from the 3D volumes. Similar to our findings, the authors found that ICCs observed for all muscle volume estimations were excellent (ICC ranging from 0.988 to 0.997). 

Most of the studies analyzing the quadriceps femoris and the hamstring muscle cross-sectional areas investigated mainly the validity, however, intra-and/or interrater reliability has rarely been reported as a part of their investigations [7]. Tate et al. conducted a study describing the lower extremity muscle morphology in 10 young athletes [13]. Unlike the 4 mm interslice distance used in our study, the authors collected 10 mm slice thickness images on the thigh. However, they reduced the slice thickness to 5 mm in the knee section, to increase the definition in this region. As part of their research, they established the intrarater reliability of muscle outline digitization from anatomic scans of 8 out of 10 participants. Similar to our findings, their results showed high reproducibility for the observer (ICC ranging from 0.95 to 0.99). 

In a recent study, Rothwell et al. investigated the application of MRI for measuring bilateral lower limb muscle size in 15 healthy males [14]. They did the measurement using slices with 15 mm interslice distance to reduce lower limb muscle volume analysis time demands and established reliability of these measurements on only two of the study participants. The authors found that the use of an interslice distance of 15 mm considerably reduced the analysis time required. Besides, between-session reliability was good for the manual measurement of muscle size from magnetic resonance images. However, their data support the application of a larger interslice distance for longer muscles and taller individuals, and measurement error was larger with increased between-session time. Muscle volume showed good between-session measurement intrarater reliability in their study, with the exception of smaller muscles in areas where boundaries can be difficult to identify, such as gluteus minimus, adductor brevis, popliteus, flexor hallucis longus, and flexor digitorum longus. In a recent review, the validity of the comparison between segmentation data from techniques using reduced number of slices and slice-by-slice segmentation was shown to vary from poor to excellent [7]. Reducing the number of slices systematically increased the error, and the number and the choice of slices to the segment were specific to each muscle [7]. 

In research and clinical contexts, manual segmentation remains the most accurate method to distinguish and predict the volume of quadriceps femoris and hamstring muscles [7,21]. In a recent systematic review, Pons et al. searched the metrological qualities of the currently used techniques to quantify skeletal muscle volume and 3D shape in healthy and pathological muscles [7]. They proposed that the reduction in number of manually segmented slices is possible either with appropriately chosen segmented slices or with the deformation of a parametric specific object (DPSO) method [7]. In a study evaluating the reproducibility of muscle reconstruction using the DPSO method, Südhoff et al. reported over 0.85 ICC for all muscles [22].

Mandic et al. explored the sensitivity of the automated technique to detect changes in quadriceps volume in response to 8 weeks of resistance training [3]. They concluded that the automated method showed an excellent correlation with manual segmentation and could detect clinically relevant magnitudes of exercise-induced muscle hypertrophy. Similarly, Le Trotter et al. used an atlas-based automatic segmentation method to quantify the volume of the quadriceps femoris muscle group [23]. They proposed the fully automated, multiatlas-based approach for global volume measurement, whereas the semiautomated, single-atlas-based approach for volume measurements of individual muscles and longitudinal investigations. Automated techniques are promising, but data is insufficient for their validation and reliability [7].

Muscle fiber composition, intracellular and extracellular water content, perfusion and lipids are the determinants of T2 relaxation time [8]. Our results showed that intra- and interobserver reliability was excellent also for T2 relaxation time measurements of quadriceps femoris and hamstring muscles, except for right quadriceps muscle which was measured by Specialist-2 (ICC: 0.74, CI: 0.27–0.93). Sun et al. investigated the difference in quadriceps femoris and the hamstring muscles between snowboarding halfpipe athletes and healthy volunteers using quantitative multiparametric MRI [8]. Similarly, the authors found the excellent interrater reliability for T2 relaxation time (ICCs ranging from 0.84 to 0.99). According to the results of a study in patients with juvenile dermatomyositis (JDM), T2 relaxation times were shown to be significantly increased in active JDM [24]. Additionally, good correlations were found between MRI scores and muscle strength and function measurements. Based on the aforementioned studies and the results we found in our research, T2 relaxation time measurement might be reliably recommended in cases where muscle inflammation will be evaluated [8,24].

The first limitation of our study is the small number of participants, however, the bilateral thigh muscles of 10 participants (a total of 40 quadriceps and hamstring muscles) were measured from axial images taken at 4 mm intervals (64 images for each muscle) twice by each sports medicine specialist. Muscle volume measurement from a total of 5120 images and T2 relaxation time measurement from a total of 80 images by each sports medicine physician is one of the strengths of our study. The second limitation of our study is that the study was conducted in young-middle age adults and the results were specific to this group. The third limitation is that we conducted this study on recreational athletes. Further studies with different age groups and training status will deepen our understanding of this field. The European Working Group on Sarcopenia in Older People also encourage more research in the field of sarcopenia in order to prevent or delay adverse health outcomes [25].

In conclusion, we evaluated the reliability and the reproducibility of volume and T2 relaxation time measurements of quadriceps femoris and hamstring muscles through images obtained using a 3.0 Tesla MRI. We found good to excellent intraobserver and interobserver reliability. Volume and T2 relaxation time measurements of quadriceps femoris and hamstring muscles with 3.0 Tesla MRI are reliable and reproducible. These measurements, which enable the quantitative evaluation of the thigh muscles, can be used in clinical practice related to follow-up of athletes, skeletal muscle diseases, and sarcopenia.

## Informed consent

Hacettepe University Clinical Research Ethics Committee approved the study with the KA-19100 protocol code and the 2019/17-13 decision number. All participants were informed about the procedure, and they provided written consent, which was in accordance with the Helsinki Declaration.

## References

[ref1] (2018). Muscle thickness correlates to muscle cross-sectional area in the assessment of strength training-induced hypertrophy. Scandinavian Journal of Medicine & Science in Sports.

[ref2] (1993). Morphometry of human thigh muscles. Determination of fascicle architecture by magnetic resonance imaging. Journal of Anatomy.

[ref3] (2020). Automated assessment of regional muscle volume and hypertrophy using MRI. Scientific Reports.

[ref4] (2021). The effect of ageing on skeletal muscle as assessed by quantitative MR imaging: an association with frailty and muscle strength. Aging Clinical and Experimental Research.

[ref5] (2010). Neuromuscular imaging in inherited muscle diseases. European Radiology.

[ref6] (2012). Imaging of muscle injury in the elite athlete. The British Journal of Radiology.

[ref7] (2018). Quantifying skeletal muscle volume and shape in humans using MRI: a systematic review of validity and reliability. PLoS One.

[ref8] (2018). Comparison thigh skeletal muscles between snowboarding halfpipe athletes and healthy volunteers using quantitative multi-parameter magnetic resonance imaging at rest. Chinese Medical Journal.

[ref9] (2018). Diagnostic imaging of osteoporosis and sarcopenia: a narrative review. Quantitative Imaging in Medicine and Surgery.

[ref10] (2019). The muscle cross-sectional area on MRI of the shoulder can predict muscle volume. Clinical Orthopaedics and Related Research.

[ref11] (2015). Assessment of lean mass and physical performance in sarcopenia. Journal of Clinical Densitometry.

[ref12] (2010). Quantitative assessment of the T2 relaxation time of the gluteus muscles in children with Duchenne muscular dystrophy: a comparative study before and after steroid treatment. Korean Journal of Radiology.

[ref13] (2006). Lower extremity muscle morphology in young athletes: an MRI-based analysis. Medicine & Science in Sports & Exercise.

[ref14] (2019). Measuring muscle size and symmetry in healthy adult males using a time-efficient analysis of magnetic resonance images. Physiological Measurement.

[ref15] (2015). Strategies to maintain skeletal muscle mass in the injured athlete: nutritional considerations and exercise mimetics. European Journal of Sport Science.

[ref16] (2016). Effects of increased muscle strength and muscle mass on endurance-cycling performance. International Journal of Sports Physiology and Performance.

[ref17] (2017). How to determine leg dominance: the agreement between self-reported and observed performance in healthy adults. PLoS One.

[ref18] (1993). Changes in lower limb muscle cross-sectional area and tissue fluid volume after transition from standing to supine. Acta Physiologica Scandinavica.

[ref19] (2018). Bilateral quadriceps and hamstrings muscle volume asymmetries in healthy individuals. Journal of Orthopaedic Research.

[ref20] (1994). Guidelines, criteria, rules of thumb for evaluating normed and standardized assessment instruments in psychology. Psychological Assessment.

[ref21] (2014). Manual segmentation of individual muscles of the quadriceps femoris using MRI: a reappraisal. Journal of Magnetic Resonance Imaging.

[ref22] (2009). 3D-patient-specific geometry of the muscles involved in knee motion from selected MRI images. Medical and Biological Engineering and Computing.

[ref23] (2016). Volume measurements of individual muscles in human quadriceps femoris using atlas-based segmentation approaches. Magnetic Resonance Materials in Physics, Biology and Medicine.

[ref24] (2004). Quantitative assessment of MRI T2 relaxation time of thigh muscles in juvenile dermatomyositis. Rheumatology.

[ref25] (2019). Sarcopenia: revised European consensus on definition and diagnosis. Age and Ageing.

